# Life Cycle Assessment of Closed-Loop Pumped Storage Hydropower in the United States

**DOI:** 10.1021/acs.est.2c09189

**Published:** 2023-08-11

**Authors:** Timothy R. Simon, Daniel Inman, Rebecca Hanes, Gregory Avery, Dylan Hettinger, Garvin Heath

**Affiliations:** The Strategic Energy Analysis Center, National Renewable Energy Laboratory, 15013 Denver West Parkway, Golden, Colorado 80401, United States

**Keywords:** pumped storage hydropower, energy storage, life cycle assessment, energy sustainability, waterpower, hydroelectric, greenhouse gas emissions

## Abstract

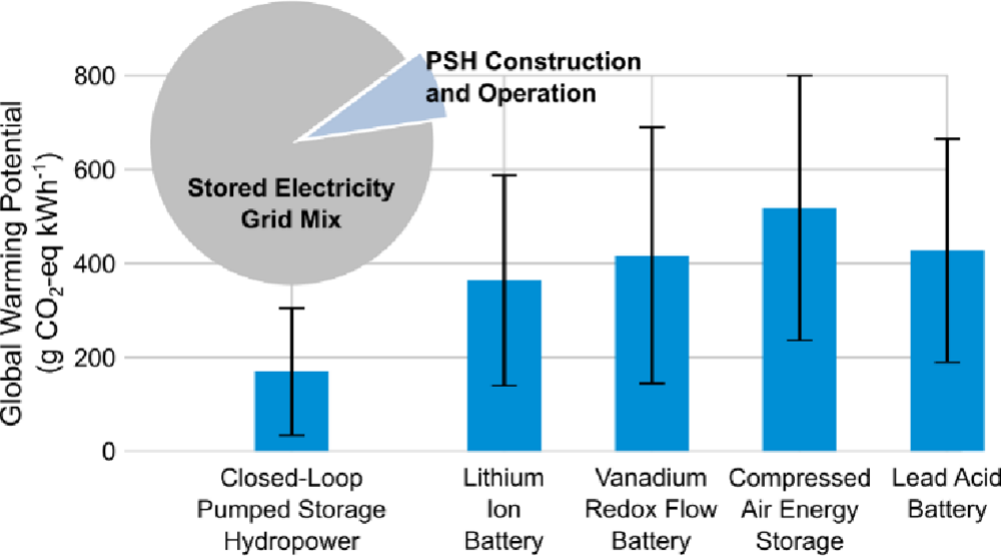

The United States has begun unprecedented efforts to decarbonize all sectors of the economy by 2050, requiring rapid deployment of variable renewable energy technologies and grid-scale energy storage. Pumped storage hydropower (PSH) is an established technology capable of providing grid-scale energy storage and grid resilience. There is limited information about the life cycle of greenhouse gas emissions associated with state-of-the-industry PSH technologies. The objective of this study is to perform a full life cycle assessment of new closed-loop PSH in the United States and assess the global warming potential (GWP) attributed to 1 kWh of stored electricity delivered to the nearest grid substation connection point. For this study, we use publicly available data from PSH facilities that are in the preliminary permitting phase. The modeling boundary is from facility construction to decommissioning. Our results estimate that the GWP of closed-loop PSH in the United States ranges from 58 to 530 g CO_2_e kWh^–1^, with the stored electricity grid mix having the largest impact, followed by concrete used in facility construction. Additionally, PSH site characteristics can have a substantive impact on GWP, with brownfield sites resulting in a 20% lower GWP compared to greenfield sites. Our results suggest that closed-loop PSH offers climate benefits over other energy storage technologies.

## Abstract 2

Grid-scale energy storage is needed to transition to a net-zero carbon economy, yet few studies compare the carbon impacts of storage technologies. Results of this study suggest that pumped storage hydropower has the lowest life cycle greenhouse gas emissions compared to other energy storage options.

## Introduction

The U.S. government enacted a long-term national strategy in 2021 to achieve net-zero carbon emissions in every sector of the economy by 2050.^1^ To meet this goal, our nation is working to electrify end uses and decarbonize electricity production, which will necessitate unprecedented deployment of renewable energy technologies. However, a fundamental bottleneck that inhibits the end use of renewable electricity is storage. The U.S. grid is built around technologies that provide inertia through synchronous generators producing an alternating current of 50 or 60 Hz.^2^ This inertia enables resiliency during system outages and failures. Technologies such as wind and solar power contribute to electricity decarbonization goals yet are temporally variable and do not provide grid inertia; therefore, they require grid-scale storage for efficient dispatching.

A Sandia National Laboratories report that categorizes available storage technologies by the services they provide (e.g., bulk energy storage, ancillary services, transmission infrastructure services, distribution infrastructure services, customer energy management services, and stacked services)^3^ and their relative maturity indicates that pumped storage hydropower (PSH) and compressed-air energy storage (CAES) are well suited for grid-scale energy storage and for providing grid inertia.^4^ At present, PSH and CAES are the only bulk energy storage technologies that have been deployed commercially: in 2019, domestic PSH had 22.9 GW of generating capacity (93% of domestic energy storage capacity) and CAES had 110 MW.^5,6^

Despite recent interest in PSH, many questions remain regarding the overall sustainability of PSH projects. Little is known about how the environmental impacts from PSH compare to those of other storage technologies and how different technology configurations may affect PSH life cycle impacts. In the context of recent climate goals, it is important to understand the relative global warming potential (GWP) of energy storage technologies to evaluate the degree to which various technologies contribute to these goals. Life cycle assessment (LCA) is an established method for comparing the GWP of competing systems and/or products and for exploring life cycle GWP impacts of process-level decisions.^7^ While PSH has been compared to other energy storage technologies in previous studies, these studies do not consider U.S.-specific conditions such as the U.S. grid mix, potential changes to the grid mix over time, advances in PSH technology, and project-level design assumptions.^2,8−11^ Using an LCA approach allows for holistic understanding of both the direct and indirect greenhouse gas (GHG) emissions, thus helping to avoid problem-shifting.^12^ This information can be used in making lower-GHG design decisions for new PSH facilities.

The objective of this study is to perform a full LCA of new closed-loop PSH in the United States and assess the GWP attributed to 1 kWh of stored electricity delivered to the nearest grid substation connection point. Additionally, we perform scenario analyses to explore various design assumptions and compare our results to published LCA results for other energy storage technologies.

## Methods

For this study, we use the Python-based Brightway2 LCA modeling framework^13^ to develop and link unit processes. Many of the processes used in this study were developed from primary, publicly available data and input from industry experts. In the absence of such data, we use ecoinvent v.3.8^14^ processes modified to reflect U.S. conditions and to account for embodied emissions and energy flows. This study follows standards for LCA from the International Organization for Standardization, including stakeholder and external reviews^15^ by experts from industry, academia, and government.

### Modeling Approach and Assumptions

**Figure 1:**
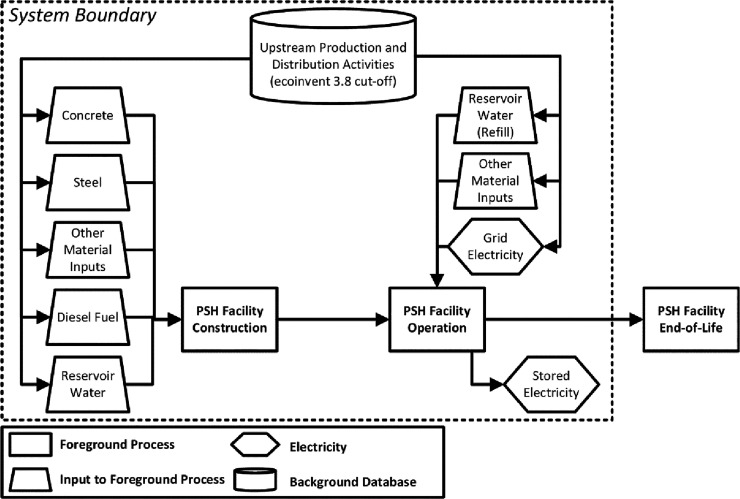
Scope and boundary used in this study.

The scope for this study is closed-loop PSH facilities in the contiguous United States and includes embodied energy and material flows (Figure 1) for facility construction, operation, and maintenance. Closed-loop PSH facilities are not continuously connected to a naturally flowing water source. The functional unit is 1 kWh of stored electricity delivered to the nearest grid substation connection point. Projected changes in the grid mix over the lifetime of the PSH facility and the associated changes in the embodied emissions of the stored electricity are based on the National Renewable Energy Laboratory’s (NREL’s) Regional Energy Deployment System (ReEDS). The ReEDS model is a publicly available long-term capacity expansion model of the U.S. electric power sector.^16^ The Base Case scenario is based on the weighted average PSH facility design from 35 proposed sites in the preliminary permitting phase (Error! Reference source not found. and Figure S1 in the SI). We used annual electricity delivered to weight the life cycle inventory (LCI) data set (eq 1), as opposed to installed capacity. Because of the weighting process, the Base Case is a representative design rather than a specific PSH facility. We use scenario analyses to explore the impact of site selection, assumed facility lifetime, electricity grid mix, and installed capacity on the estimated life cycle GWP. The complete set of LCI data used in this study is available from the authors on request.

The following sections summarize the data sources, methods, and assumptions employed in the modeling of each life cycle stage considered.

### PSH Facility Construction

The construction stage includes material and energy inputs for all new construction that will be required to yield a fully operational closed-loop PSH storage plant (Figure S2 in the SI). For sites that do not already have access to an existing reservoir(s), the construction phase includes excavation of an upper and/or lower reservoir; construction of dams, penstocks, roads, powerhouse, and electricity transmission infrastructure; production and installation of generation equipment; and diesel and electricity use by construction equipment and vehicles. For sites with reservoir access, the construction of the reservoir(s) was excluded from the construction phase. The primary materials used in construction are concrete, sand and gravel (used in dam construction), steel, stainless steel, and copper. Transportation of these materials to the construction site is also included.

### Operation and Maintenance

We account for all inputs that are required to operate and maintain the PSH facility over the course of its assumed 80-year lifetime. This includes required facility maintenance and upkeep, replacement of equipment every 40 years, the initial water fill and annual refill for the reservoir,^17^ electricity used to supply water fill and refill to the reservoir, electricity grid mix to be stored, and GHG emissions attributed to newly constructed reservoirs, which were calculated from published emission rates.^18^ Existing reservoirs used in brownfield PSH sites were assumed to produce no net GHG emissions.

### End of Life

For the Base Case, we assume that the PSH facility will be abandoned and left intact and all maintenance will be discontinued. Although we do not assess demolition as an end-of-life scenario in this study, one can assume that demolition will substantially increase the life cycle GHG emissions. Future work should examine the life cycle impacts of various decommissioning options, which are summarized in the SI.

### LCI Data

This study is based on 35 closed-loop PSH sites that are currently in the preliminary permitting stage (Table S1 and Figure S1 in the SI). Four of the 35 sites have detailed alternative designs, for a total of 39 preliminary PSH designs. At the time of our LCI data collection, all the sites used had yet to begin construction; thus, we relied on publicly available data.

For each LCI input, the value used as an input or output flow is a weighted average from the sites that have data listed or that can be calculated from available metrics and other specifications:
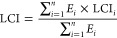
1where LCI = life cycle inventory input, *E_i_* = estimated electricity delivered from the *i*th storage plant annually, LCI_*i*_ = life cycle inventory input for the *i*th site, and *n* = total number of PSH sites contributing data to LCI.

Rather than weighting the average LCI input and output flows by installed capacity, the inventory data were weighted by annual electricity delivered, which we feel is a more accurate representation of the system’s functionality. This method was used to calculate the initial construction inputs as well as annual inputs required in a typical year of operation. The combined impact of GHG emissions is calculated using the International Panel on Climate Change Global 100-year Warming Potential (IPCC 100a GWP) for all gases.^19^
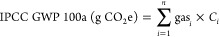
2where *C_i_* = carbon intensity for the *i*th gas, g = grams, and CO_2_e = carbon dioxide equivalents based on the IPCC 100a weighting.

### Comparative Technologies

In addition to assessing the life cycle GWP from closed-loop PSH in the United States, we also collected literature data from published LCA studies on other energy storage technologies. Storage technologies considered for comparison include CAES, utility-scale lithium-ion batteries (LIBs), utility-scale lead-acid (PbAc) batteries, and vanadium redox flow batteries (VRFBs). A detailed list of all comparative storage technologies considered in this study and associated references are provided in Table S2 in the SI. The values for alternative technologies are used as a basis for comparing the results of our LCA against results from comparative energy storage systems. For the purposes of our comparisons, we harmonize previous LCA results based on the functional unit reported and assumptions regarding the source of stored energy. That said, the grid mixes assessed in this study are commensurate with but not identical to those reported in previous studies. We use results from the ReEDS model to inform our assumptions regarding grid mix over time. In contrast, previous LCA studies generally rely on static country/region-specific grid mix information.

### Scenario Analysis

Including the Base Case, we evaluate 17 scenarios in this study (Table 1). The scenarios examine the impact on the life cycle GWP of (1) facility lifetime (80 vs 100 years), (2) installed capacity, (3) whether the proposed site is greenfield or brownfield, (4) reservoir liner material, and (5) the stored electricity grid mix. In each scenario, only the parameters mentioned in the Description are varied; all other parameters remain at Base Case values.

**Table 1:** Scenarios Evaluated in This Study

name	notation	description
Base Case	BC	835 MW, 2060 GWh/year delivered, geomembrane, renewable electricity stored, 80-year lifetime
100-Year Lifetime	100-Yr	100-year lifetime
Installed Capacity: Small	Small	installed capacity of less than 500 MW
Installed Capacity: Medium	Med	installed capacity between 500 and 1000 MW
Installed Capacity: Large	Large	installed capacity greater than 1000 MW
Site: Greenfield	GF	facility is built on a greenfield site
Site: Brownfield	BF	facility is built on a brownfield site
Liner: No Liner	No Liner	no liner is used
Liner: Clay Liner	Clay	clay liner is used
Liner: Concrete Liner	Concrete	concrete liner is used
Liner: Asphalt Liner	Asphalt	asphalt liner is used
Grid Mix: Mid-Case 80	Mid-80	grid mix is the mid-case ReEDS scenario
Grid Mix: Mid-Case 100	Mid-100	grid mix is the mid-case ReEDS scenario and system lifetime is extended to 100 years
Grid Mix: 95% by 2035 80	95x35-80	grid mix reflects a 95% reduction of CO_2_ emissions from the power sector by 2035 and 100% reduction of CO_2_ emissions by 2050
Grid Mix: 95% by 2035 100	95x35-100	grid mix reflects a 95% reduction of CO_2_ emissions from the power sector by 2035 and 100% reduction of CO_2_ emissions by 2050. System lifetime is extended to 100 years
Grid Mix: 95% by 2050 80	95x50-80	grid mix reflects a 95% reduction of CO_2_ emissions from the power sector by 2050
Grid Mix: 95% by 2050 100	95x50-100	grid mix reflects a 95% reduction of CO_2_ emissions from the power sector by 2050. System lifetime is extended to 100 years

### Base Case Scenario

The Base Case scenario represents the average closed-loop PSH facility that is under preliminary permitting in the United States and the state of the industry as of 2022 in terms of technology and facility design. In our Base Case scenario, we assume the stored electricity will be entirely generated by renewable technologies, including concentrating solar power, photovoltaics, and both onshore and offshore wind (Figure S3 in the SI). For this, we used NREL’s ReEDS model to project the anticipated power grid mix over an 80-year time frame.^20^ Previous LCAs performed on energy storage technologies have shown that the grid mix used to generate stored electricity has a substantial impact on the overall life cycle GHG emissions.^8,9^ The stored grid mix is dependent on both location and the storage application. There are four primary applications of grid storage summarized by Baumann et al.:^21^ electric time shift, increase of photovoltaics self-consumption, primary regulation, and renewables support. Both electric time shift and primary regulation would include a grid mix of renewable and nonrenewable fossil fuel resources, whereas photovoltaics self-consumption and renewables support would only contain renewable energy technology mixes. We assume that the most applicable uses of PSH are electric time shift, primary regulation, and renewables support. For this reason, we evaluate both renewable and nonrenewable energy technology grid mixes in the Electricity Grid Mix scenarios, described below, that account for generation over the projected lifetime of the plant.

### 100-Year Lifetime Scenario

We assume that the lifetime of a typical closed-loop PSH facility is 80 years. However, some sources estimate the lifetime to be over 100 years.^8−11,22−30^ The 100-Year Lifetime scenario evaluates the impact of extending the lifetime of a PSH plant from 80 to 100 years. The primary difference between the Base Case and the 100-Year Lifetime scenario is the number of equipment replacements, which we assume will occur every 40 years. This impacts both copper and steel inputs, as well as the additional electricity delivered from the plant. In addition to this scenario, we jointly evaluate the 100-year lifetime assumption as part of the Electricity Grid Mix scenarios discussed below.

### Installed Capacity Scenarios

The installed capacities of permitted sites range from 50 to 3600 MW. While it is fair to assume the construction of larger plants has a considerably greater overall impact, the GWP per functional unit also varies due to the differences in estimated electricity delivered over the life of the plant (177–7900 GWh for all sites considered (Table S1)). In the Installed Capacity scenarios, sites are binned into three categories: *Small* sites have installed capacities of less than 500 MW; *Medium* sites have installed capacities between 500 and 1000 MW; and *Large* sites have installed capacities greater than 1000 MW. This allows for a comparative LCA pertaining to the size of the PSH installation.

### Greenfield and Brownfield Scenarios

PSH facility siting is geographically limited because of reservoir head height requirements. The majority of proposed PSH sites are in areas that have high topographic relief. One major consideration for PSH construction sites is whether it is a greenfield or brownfield site.^31^ Sites constructed with required development on vacant land are considered greenfield; brownfield PSH sites tend to utilize old mining grounds with preexisting quarries or reservoirs. Out of all sites contributing data to the LCI, 8 are brownfield and 27 are greenfield (or 31 when including site alternatives).

### Reservoir Liner Material Scenarios

Newly constructed PSH reservoirs may use a liner to prevent seepage into the ground. The most common liner used for PSH reservoir construction is a geomembrane liner constructed from woven polymer composites.^32^ We assume a geomembrane liner in our Base Case scenario. We also evaluate the following liner options: no liner, clay, concrete, and asphalt. Cost and location are the biggest considerations when determining which liner to install on a site. It should be noted that none of the 39 proposed PSH designs used in this study specify the type of liner material to be used.

### Electricity Grid Mix Scenarios

We used NREL’s ReEDS model to simulate the stored grid mix over the life of the plant under three different grid mixes and two facility lifetimes. For this study, we use projected grid mixes from three standard ReEDS scenarios: the Mid-Case, 95% by 2035, and 95% by 2050. The Mid-Case 80 and Mid-Case 100 scenarios use default ReEDS assumptions without any new carbon policies in place for 80- and 100-year ReEDS model simulations. The 95% by 2035 ReEDS scenario assumes that CO_2_ emissions will decrease linearly to 95% below 2005 levels by 2035 and to 100% by 2050. The 95% by 2050 ReEDS scenario assumes that CO_2_ emissions will decrease linearly by 95% below 2005 levels by 2050. Figures S4–S6 in the SI illustrate the technology ratios of the time-varying grid mixes over an 80-year lifetime. Previous literature contains more information on the ReEDS model and the standard scenarios.^20,33^

The present study does not account for carbon capture and sequestration additions to biopower, coal, or natural gas power plants. Additionally, emission factors for various technologies are taken from the ecoinvent 3.8 database rather than ReEDS, which only accounts for carbon dioxide, methane, nitrous oxide, and sulfur dioxide emissions. All grid mix scenarios are considered for system lifetimes of 80 and 100 years. The renewable grid mix is assumed in the Base Case, 100-Year Lifetime, Small, Medium, Large, Greenfield, Brownfield, No Liner, Clay Liner, Concrete Liner, and Asphalt Liner scenarios. Full grid mix scenarios include the Mid-Case 80, Mid-Case 100, 95x35-80, 95x35-100, 95x50-80, and 95x50-100 scenarios (Table 1).

## Results and Discussion

**Figure 2:**
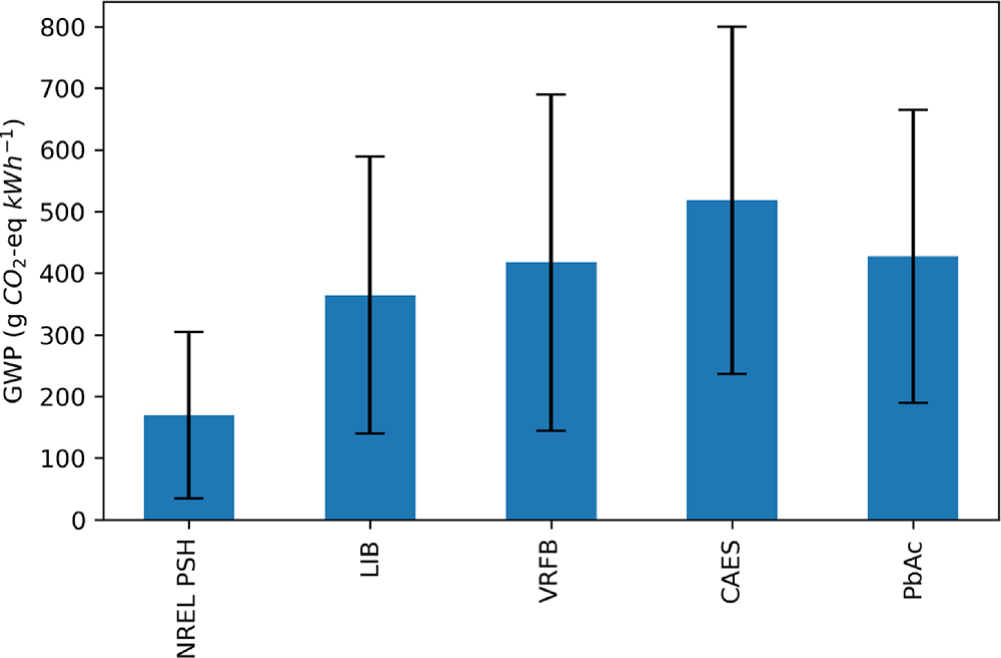
Global warming potential (GWP) of closed-loop PSH (NREL PSH), calculated in this study, compared to literature GWP values for lithium-ion battery storage (LIB), vanadium redox flow batteries (VRFB), compressed-air energy storage (CAES), and lead-acid battery energy storage (PbAc). The bar heights indicate the mean GWP for each technology, and the error bars indicate the GWP standard deviation.

The mean and standard deviation GWP from all scenarios evaluated are presented and compared to other storage technologies in Figure 2. Overall, the PSH scenarios evaluated in this study result in a lower GWP on a functional unit basis than all other storage technologies evaluated in the literature. Results from this study suggest that the GWP of closed-loop PSH is between 58 and 530 g CO_2_e kWh^–1^, with the Base Case GWP being 86 g CO_2_e kWh^–1^. Across scenarios and technologies evaluated, the source of stored electricity has the largest impact on the GWP of electricity storage technologies. For PSH, facility-level decisions such as liner type, assumed facility lifetime, whether the facility is built on a greenfield or brownfield site, and installed capacity have small impacts on the GWP compared to the assumed grid mix of the stored energy. It is important to note that the stored grid mix in comparative studies, both renewable and full grid mix, do not directly align with the assumptions used in this study. The comparative technologies used in this study are based on values from the literature, and the assumed grid mixes for these studies vary. Scenario-specific results are presented and discussed in more detail in the Scenario Analysis section.

### Scenario Analysis

In the following section, we present GWP results from the scenario analyses performed and compare those to results from the literature for other energy storage technologies.

### Base Case

**Figure 3:**
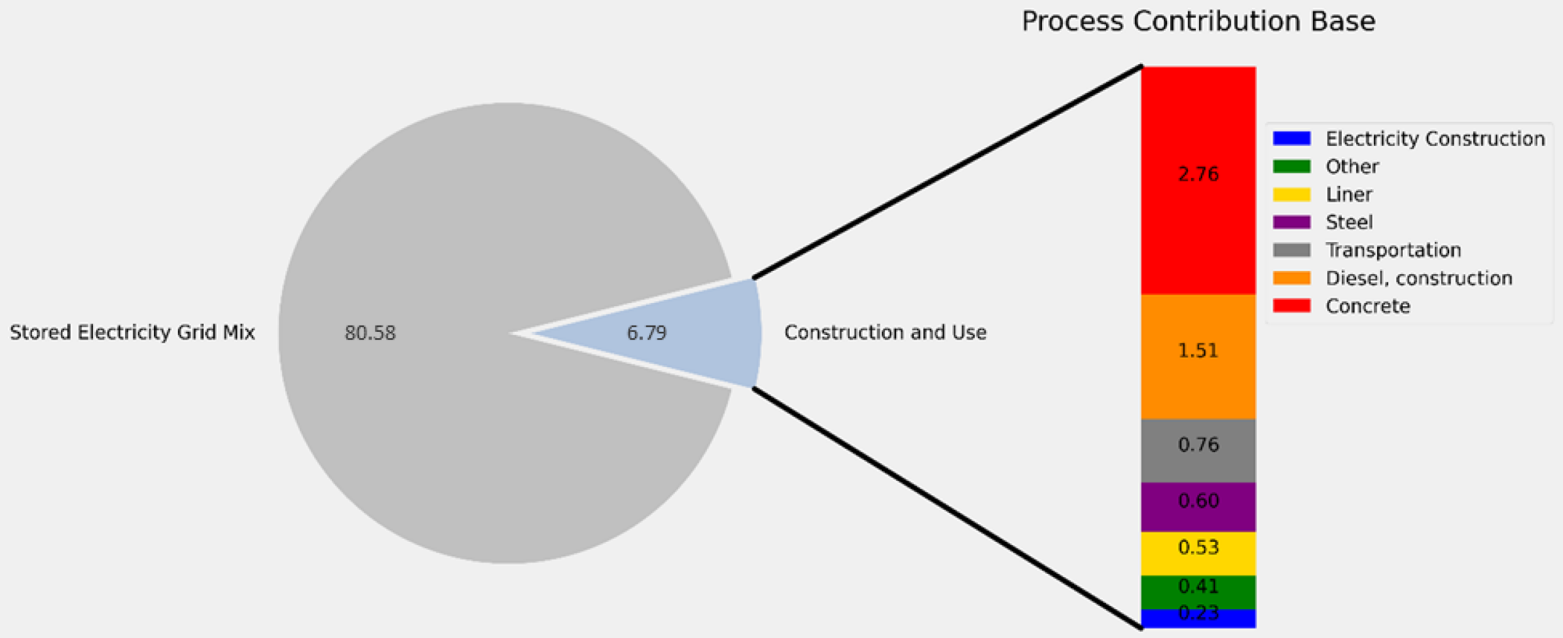
Life cycle 100-year GWP for the Base Case scenario, disaggregated according to contributions from the primary life cycle phases.

Results of the Base Case are presented in Figure 3. Overall, the GWP of 1 kWh of electricity delivered by the closed-loop PSH system to the nearest grid substation connection point is estimated to be 86 g CO_2_e kWh^–1^. In terms of contribution to the overall GWP, the emissions from the source of stored electricity account for the majority of GWP. This is consistent with results reported elsewhere in the literature.^8,9^ The second largest source of emissions is from the construction phase. Concrete and steel used during construction account for ∼4% of the GWP. Diesel fuel used by on-site heavy equipment, transport of materials to the construction site, and installation of a geomembrane liner account for less than 5% of the GWP.

Previously reported values for life cycle GWP of PSH vary widely. Estimates range from 5.6 g CO_2_e kWh^–1^ to more than 650 g CO_2_e kWh^–1^.^2,9,28^ The large variance in GWP estimates from PSH can be attributed, in part, to variable assumptions in the plant lifetime, plant capacities, data provenance (e.g., actual operating facilities^2^ vs simulated facilities,^8,9,25^), facility type and vintage (e.g., open vs closed-loop), facility location, and assumptions regarding the source of electrical energy being stored. Results of our Base Case are commensurate with those reported in the literature with similar LCA assumptions regarding the source of stored electricity. Oliveira et al.^8^ report GWP from PSH to be ∼100 and <50 g CO_2_e kWh^–1^ for electricity stored from photovoltaic and wind power sources, respectively. Similarly, Abdon et al.^9^ report estimated GWP to be between ∼50 and 150 g CO_2_e kWh^–1^ for PSH storing wind-derived electrical energy.

### Installed Capacity

**Figure 4:**
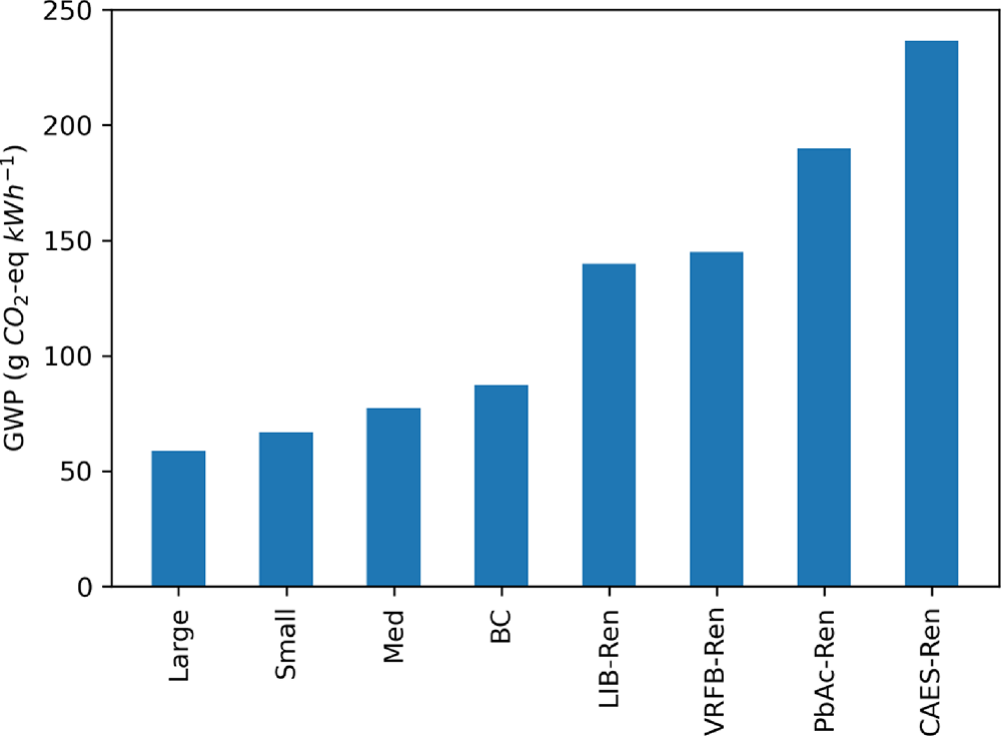
Impact of installed capacity on the life cycle GWP of closed-loop PSH facilities. The Base Case results reflect an installed capacity of 835 MW. Small sites have installed capacities of less than 500 MW; Medium sites have installed capacities between 500 and 1000 MW; and Large sites have installed capacities greater than 1000 MW. The annual electricity delivered for Small, Medium, and Large PSH facilities is 850, 1394, and 4229 GWh yr.^–1^, respectively.

The impact of varying the installed capacity on the life cycle GWP is presented in Figure 4. On a functional unit basis, the impact of economies of scale on GWP are evident when comparing the Small (65 g CO_2_e kWh^–1^) and Large (58 g CO_2_e kWh^–1^) PSH sites. However, the Medium case does not follow this trend. For the Medium case, the results are somewhat biased because the installed capacities and annual electricity delivered for facilities in this bin do not follow a linear trend. With a functional unit of 1 kWh of electricity delivered, the results rely on the estimated annual electricity delivered over the lifetime of the system. The average annual electricity delivered for the Base Case, Small, Medium, and Large PSH facilities are 835, 850, 1394, and 4229 GWh yr.^–1^, respectively. A plant with higher installed capacity will require proportionally scaled inventories for several LCI inputs, but if the consequential increase in delivered electricity does not scale uniformly with capacity, the overall impacts will likewise not scale uniformly with capacity. That said, when evaluating the GWP over the lifetime of the system, the results do align with expected results from economies of scale. Across the installed capacities evaluated, the GWP of closed-loop PSH varies from 58 to 86 g CO_2_e kWh^–1^, with the large PSH facilities (mean installed capacity of 4229 GWh yr.^–1^) having the lowest GWP on a functional unit basis.

### Site Conditions

**Figure 5:**
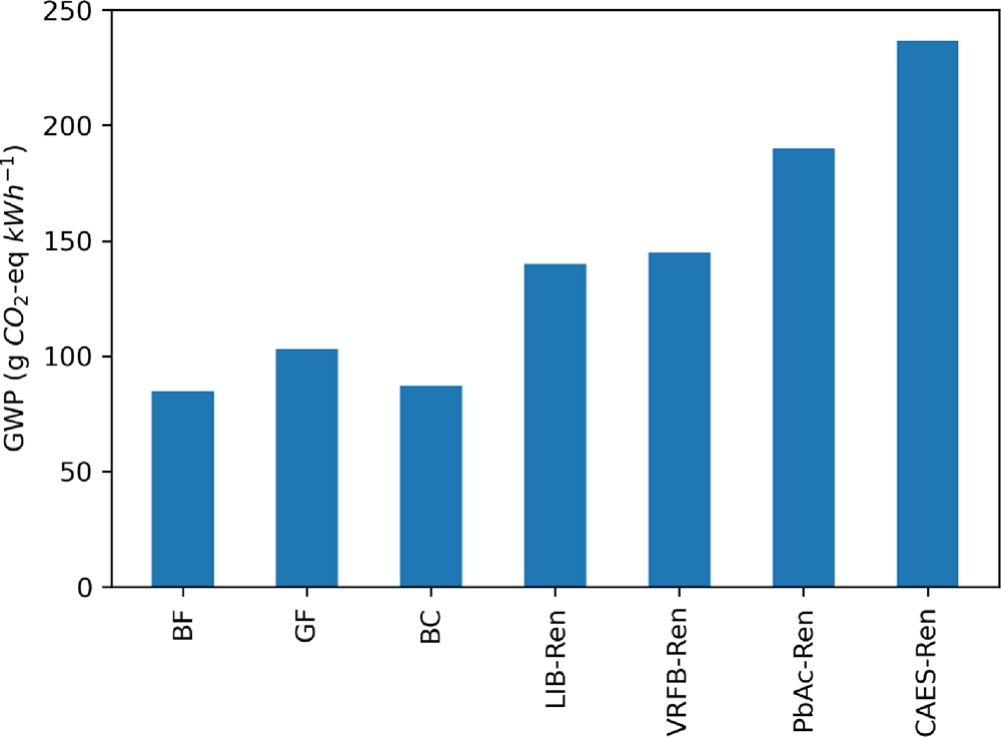
Impact of the site conditions on the life cycle GWP of closed-loop PSH facilities. Results of our Base Case compared to PSH facilities built on greenfield (GF) (undeveloped) sites and brownfield (BF) (previously developed) sites.

Results of the Base Case are compared to the greenfield and brownfield sites in Figure 5. The greenfield sites have a higher GWP than brownfield sites, which do not require the excavation of one reservoir. On a functional unit basis, greenfield sites are estimated to emit approximately 30% more GHGs than brownfield sites. The Base Case sites have lower emissions than the greenfield sites due to the weighting involved: the Base Case LCI represents a weighted average site, with annual electricity delivered as the weights. Although there were fewer brownfield sites than greenfield, the brownfield sites generally had larger annual electricity delivered values; this leads to the Base Case emissions more closely resembling the brownfield sites. These results suggest that brownfield sites are favorable for reducing GWP in the siting of new PSH facilities. That said, we have not performed any costing, environmental impact, or geospatial resource availability analyses, all of which should be considered when siting a potential PSH facility.

### Liner Material Options

**Figure 6:**
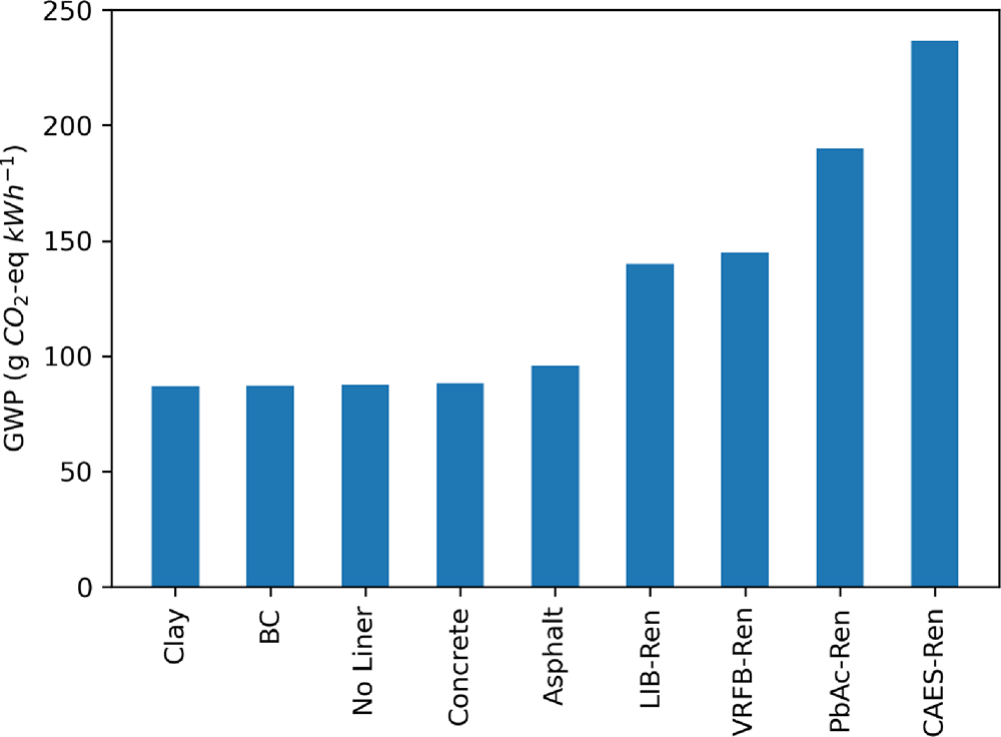
Impact of reservoir liner material on the life cycle GWP of closed-loop PSH facilities. The Base Case results reflect the use of a geomembrane liner.

New PSH facilities may use a liner to prevent leakage from their upper and lower reservoirs. The choice of liner material depends on local soil and geological conditions, and the permit data collected for this study did not specify liner materials. The impact of reservoir liner material on the life cycle GWP is shown in Figure 6 for four commonly used materials: geomembrane (Base Case), asphalt, concrete, and clay. We did not assume any maintenance or replacement of the reservoir liners over the course of the facility’s 80-year lifetime. Other than asphalt, the choice of material used to line the reservoir has little impact on the life cycle GWP of a closed-loop PSH facility on a functional unit basis. The variance in GWP across the four liner options assessed in this study was less than 9 g CO_2_e kWh^–1^.

### Electricity Grid Mix

**Figure 7:**
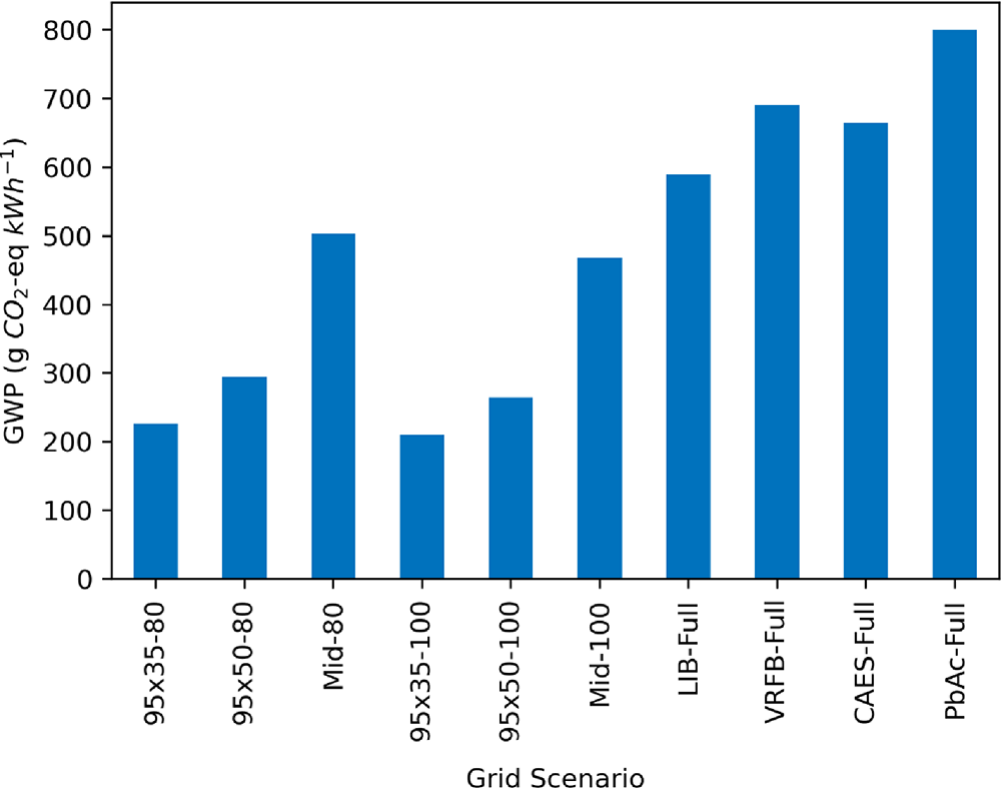
Impact of full grid mix ReEDS scenarios on the life cycle global warming potential of closed-loop PSH and four alternative energy storage technologies: lithium-ion batteries (LIB), vanadium redox flow batteries (VRFB), compressed air energy storage (CAES), and lead-acid batteries (PbAc).

The impact of varying the stored grid mix on the GWP of PSH is shown in Figure 7. As reported in other LCA studies, the grid mix of the electricity being stored by the PSH facility has the single largest impact on the life cycle GWP. By changing the stored electricity in the Base Case from a renewables-only grid mix to a full grid scenario, the GWP increases sixfold, from 86 to 530 g CO_2_ kWh^–1^. When comparing across other storage technologies and assuming the stored electricity is from more fossil-fuel-dominated grid mixes, PSH results in the lowest GWP on a functional unit basis, followed by LIB, VRFB, CAES, and PbAc.

### Implications

Results from this study suggest that closed-loop PSH can offer climate benefits over other energy storage technologies. Compared to data from the literature on other energy storage technologies, closed-loop PSH has a lower GWP than all other energy storage technologies evaluated in this study. Based on our scenario analysis, the source of stored electricity is the predominant factor impacting the GWP of PSH. This study also found that certain project-level decisions can have a substantive impact on GWP. Constructing a new closed-loop PSH facility on a brownfield as opposed to a greenfield site can result in a 20% lower GWP. Similarly, taking advantage of economies of scale can have a positive impact on life cycle GWP, with larger facilities having a lower GWP than smaller ones. In contrast, the choice of reservoir liner material and anticipated facility lifetime have marginal impacts on the life cycle GWP of closed-loop PSH.

Decarbonizing the electrical grid in the United States will require grid-scale energy storage options that minimize additional carbon emissions. Our results suggest that closed-loop PSH is a promising energy storage option in terms of its life cycle GHG emissions and can play a key role toward meeting our nation’s climate goals. This study did not evaluate deconstruction as a potential scenario. Further work is needed to understand the implications of various end-of-life scenarios on the GWP of closed-loop PSH.

## Supporting Information Available

Pumped storage hydropower (PSH) sites used in the life cycle inventory phase of this study; the locations of the 35 preliminary pumped storage hydropower (PSH) sites used in this study; typical closed-loop pumped storage hydropower facility; discussion of pumped storage hydropower end-of-life options; comparative storage technologies used in this study; comparative storage technologies overview; Renewable Energy Deployment System (ReEDS) Grid Mix scenarios; Projected Base Case electricity grid mix from the ReEDS model; ReEDS results for generation technology grid mix ratios over an 80-year lifetime based on the Mid-Case 80 scenario; ReEDS results for generation technology grid mix ratios over an 80-year lifetime based on the scenario for 95% CO2-eq reduction from 2005 levels by 2050; ReEDS results for generation technology grid mix ratios over an 80-year lifetime based on the scenario for 95% CO2-eq reduction from 2005 levels by 2035 and 100% CO2-eq reduction by 2050; and material inputs (PDF)
