# Supplementary material for: Life Cycle Assessment of Closed-Loop Pumped Storage Hydropower in the United States
Source: Environ Sci Technol. 2023 Aug 11;57(33):12251–8. doi: 10.1021/acs.est.2c09189 (PMC10448748; doi:10.1021/acs.est.2c09189)
Supplement: Supplementary file 1 — es2c09189_si_001.pdf [file es2c09189_si_001.pdf]

SUPPORTING INFORMATION

Life Cycle Assessment of Closed-Loop Pumped Storage Hydropower in the United States

Timothy R. Simon[1], Daniel Inman[1], Rebecca Hanes[1], Gregory Avery[1], Dylan Hettinger[1], Garvin Heath[1]*

[1] The Strategic Energy Analysis Center, National Renewable Energy Laboratory, 15013 Denver West Parkway, Golden, CO 80401

*Corresponding author: daniel.inman@nrel.gov

Number of pages: 19

Number of tables: 2

Number of figures: 6

Table S1. Pumped Storage Hydropower (PSH) Sites Used in the Life Cycle Inventory Phase of This Study

State	Site Name[a]	Capacity (MW)	Electricity Delivered (GWh yr^{-1})	Site Status	References
Arizona	Big Chino Valley	2,000	4,614	Greenfield	(*1*)
Arizona	Big Canyon	3,600	7,900	Greenfield	(*2*)
Arizona	Salt River Indian Springs	1,500	3,285	Greenfield	(*3*,*4*)
Arizona	JD Sky	800	311	Greenfield	(*5*,*6*)
Arizona	Delaney PS	200	864	Greenfield	(*7*)
Arizona	Gila River Indian Comm PS	2,100	3,504	Greenfield	(8)
Arizona	Casa Grande PS	200	864	Greenfield	(*9*)
Arizona	Sacaton	160	400	Brownfield	(*10*)
California	San Vicente	500	1,300	Greenfield	(*11*,*12*)
California	Eagle Crest	800	4,205	Brownfield	(*13*)
California	Bison Peak	330	788	Greenfield	(*14*)
California	Bison Peak (alternative 1)	360	963	Greenfield	(*14*)
California	Bison Peak (alternative 2)	360	1051	Greenfield	(*14*)
Colorado	Craig-Hayden PS	600	1,051	Greenfield	(*15*)
Idaho	Cat Creek	720	1,965	Greenfield	(*16*,*17*)
Montana	Gordon Butte	400	1,300	Greenfield	(*18*,19)
Nevada	Rose Creek	250	548	Greenfield	(*20*,*21*)
Nevada	Eldorado	600	1,051	Greenfield	(*22*)
Nevada	Eldorado (alternative)	750	1,314	Greenfield	(*22*)
Nevada	Blue Diamond PS	450	1,643	Greenfield	(*23*,*24*)
Nevada	Ruby Hill PS	200	730	Brownfield	(*25*)
New Mexico	Beclabito	1,800	2,628	Greenfield	(*26*)
New Mexico	Sweetwater PS	600	1,051	Greenfield	(*27*)
New York	Lyon Mountain Energy Storage	240	421	Brownfield	(*28*,*29*)
New York	Mooresville	49	178	Greenfield	(*30*)
Ohio	New Summit	1,500	2,000	Brownfield	(*31*)
Ohio/Kentucky	Maysville	500	876	Brownfield	(*32*)
Oklahoma	SE Oklahoma	1200	4,368	Greenfield	(*33*,*34*)
Oklahoma	Pushmataha County PS	1,200	4,368	Greenfield	(*35*)
Oklahoma	Pushmataha County PS (alternative)	1,200	4,368	Greenfield	(*35*)
Oregon	Prineville	200	525	Greenfield	(*36*–*38*)
Oregon	Swan Lake North	393	1,187	Greenfield	(*39*)
Oregon	Owyhee PS	600	946	Greenfield	(*40*,*41*)
Pennsylvania	Silver Creek	250	785	Brownfield	(*42*)
Pennsylvania	Packer-Banks PS	400	1,680	Brownfield	(*43*)
Utah	Flat Canyon PS	300	525	Greenfield	(*44*)

Washington	Goldendale	1,200	5,256	Greenfield	(*45*,*46*)
Washington	Badger Mountain PS	500	823	Greenfield	(*47*)
Wyoming	Seminoe	700	1,840	Greenfield	(*48*)

[a] All sites are under the preliminary permitting phase.

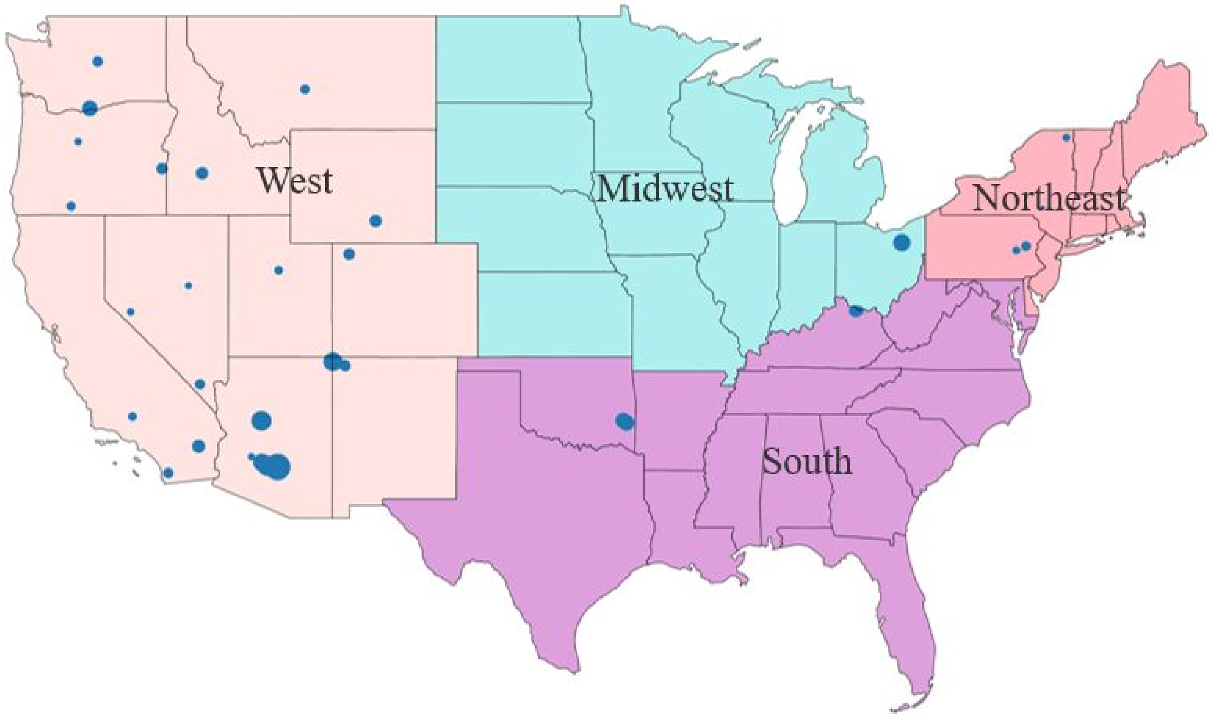

Figure S1. The locations of the 35 preliminary pumped storage hydropower (PSH) sites used in this study. Blue circles represent the approximate location of the proposed PSH facility; circles are sized based on the proposed storage capacity. Four of the sites have detailed alternative facility designs that are included in this study for a total of 39 PSH designs.

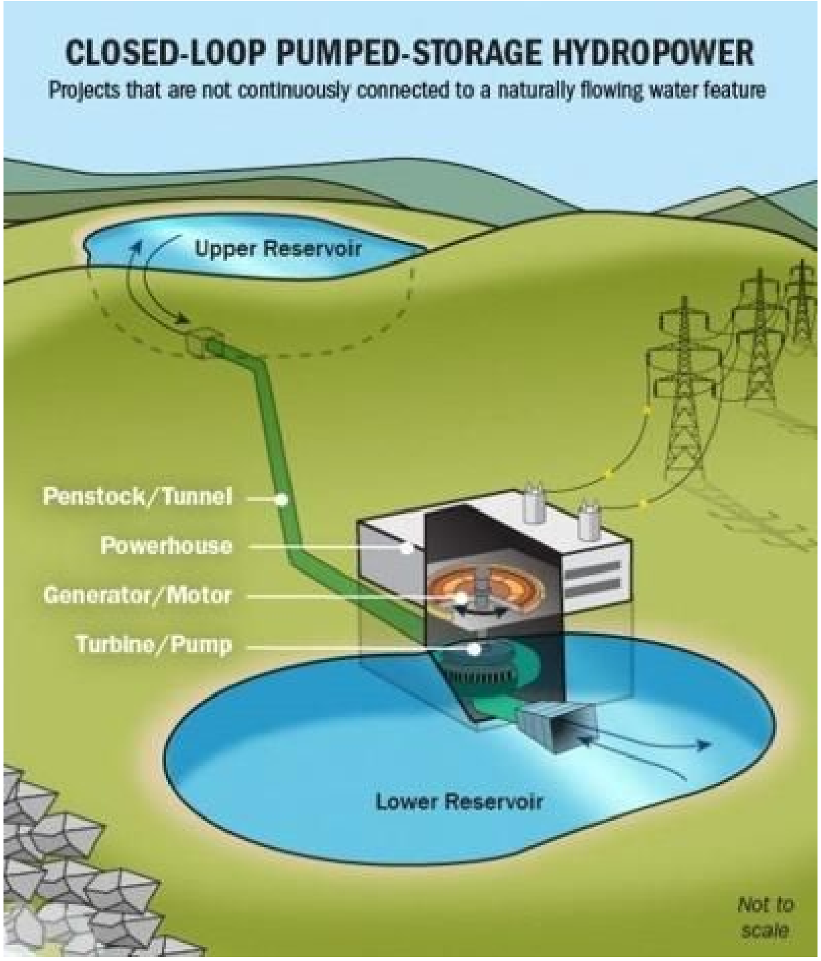

Figure S2. Typical closed-loop pumped storage hydropower facility. The construction phase in this study includes the installation of all components shown.

Discussion of Pumped Storage Hydropower End-of-Life Options

Determining an average end-of-life scenario for a typical pumped storage hydropower (PSH) facility is difficult because of a lack of data. In general, PSH facilities are expected to last 80 to 100 years. Given that most of the U.S. PSH storage capacity was built in the 1970s, we have not reached the end-of-life stage for enough facilities to make an informed generalization as to the typical fate of a PSH facility following its useful lifetime. A study by Pohl[49] analyzed trends in dam removal in the United States. The study found that from the 1980s through the 2000s the rate of dam removal in the Unites States increased, and that smaller dams were more likely to be razed than larger ones. Additionally, the study found that environmental concerns were the main impetus for dam removal, followed by safety concerns.[49] Although dam removal is becoming more common for conventional hydropower projects,[50] the rationale for removal may not be entirely applicable to closed-loop PSH facilities. In the United States, closed-loop PSH facilities do not interfere with or alter natural waterways and thus may not be viewed as being as

environmentally deleterious. Therefore, the pressure and support for the demolition of closed-loop PSH facilities following their use phase may not be as strong as it has been for conventional hydropower plants. However, other issues such as safety concerns will likely factor into the decision to demolish a PSH facility following its lifetime, and we can expect that some fraction of closed-loop PSH facilities will be completely razed following their use phase.

Table S2. Comparative Storage Technologies Used in This Study

Compared Storage Technology	Technology Abbreviation	Technology Description	Data References
Utility-Scaled Lithium-Ion Batteries: Renewables	LIB-Ren	Utility-scaled lithium-ion batteries, including lithium iron phosphate/lithium titanate, lithium manganese oxide, nickel cobalt aluminum oxide, and nickel cobalt manganese oxide. Data are taken from studies incorporating renewable energy technologies as stored grid mix.	(*51–54*)
Utility-Scaled Lithium-Ion Batteries: Full Grid Mix	LIB-Full	Utility-scaled lithium-ion batteries, including lithium iron phosphate/lithium titanate, lithium manganese oxide, nickel cobalt aluminum oxide, and nickel cobalt manganese oxide. Data taken from studies incorporating full technology grid mix including fossil fuel technologies as stored grid mix.	(*51–55*)
Vanadium Redox Flow Batteries: Renewables	VRFB-Ren	Vanadium redox flow batteries. Data taken from studies incorporating renewable energy technologies as stored grid mix.	(*51–53*)
Vanadium Redox Flow Batteries: Full Grid Mix	VRFB-Full	Vanadium redox flow batteries. Data taken from studies incorporating full technology grid mix including fossil fuel technologies as stored grid mix.	(*51–53*,*55*)
Lead-Acid Batteries: Renewables	PbAc-Ren	Lead-acid batteries. Data taken from studies incorporating renewable energy technologies as stored grid mix.	(*51–53*)
Lead-Acid Batteries: Full Grid Mix	PbAc-Full	Lead-acid batteries. Data taken from studies incorporating full technology grid mix including fossil fuel technologies as stored grid mix.	(*51–53*,*55*)
Compressed-Air Energy Storage: Renewables	CAES-Ren	Nonadiabatic compressed-air energy storage. Data taken from studies incorporating full technology grid mix including fossil fuel technologies as stored grid mix.	(*52*,*54*,*56*)

Compressed-Air Energy Storage: Full Grid Mix	CAES-Full	Nonadiabatic compressed-air energy storage. Data taken from studies incorporating full technology grid mix including fossil fuel technologies as stored grid mix.	(*52*,*54)*

Comparative Storage Technologies Overview

Compressed air energy storage (CAES) relies on coordinated operation with a conventional natural gas turbine. Cool compressed air that has been stored is heated and led through an expansion turbine to generate electricity when needed. Primary components include the compressor and motor, storage cavern, heat exchanger, and turbine/generator. The estimated system lifetime is around 30 years, and the effective efficiency is about 85%.[57] There are few large-scale examples currently in operation, but systems can be integrated to support large utilities and are seen as a promising large-scale storage system.

Lithium-ion batteries (LIBs), one of the newer technologies considered, are currently used in applications such as light-duty vehicles, consumer electronics, and industrial equipment. Interest has increased in LIBs as a promising technology for stationary energy storage. Relative to other comparable battery technologies, LIBs have high energy efficiency, long cycle life, and high energy density.[58] LIBs offer flexible installation, modularization, rapid response, and short construction cycles. Kebede et al.[59] report that LIBs have a specific energy of 75–250 Wh kg^{-1}, specific power of 150–315 W kg^{-1}, round-trip efficiency of 85%–95%, and a service lifetime of 5–15 years. Although LIBs are not promising as a high-capacity bulk energy storage technology (energy storage rating of 100 MWh), they are seen as the most promising for capacity-firming and time-shift functions.[59]

Lead-acid (PbAc) battery technology is commercially mature and well understood. PbAc batteries have a specific energy of 30–50 Wh kg^{-1}, specific power of 75–300 W kg^{-1}, round-trip efficiency of 70%–80%, and a service lifetime of 5–15 years.[59] Relative to other battery technologies, PbAc is less expensive but has lower power density, higher weight, and lower cycle life.[60] The estimated lifetime of PbAc batteries ranges from 5 to 12 years with potential extension via cell replacement.[61] Like LIBs, PbAc batteries are envisaged for capacity-firming and time-shift functions as opposed to high-capacity bulk energy storage applications.

Vanadium redox flow batteries (VRFBs) employ chemical reduction and oxidation reactions to store energy in a vanadium sulfuric acid electrolyte solution. An oxidation reaction releases electrons on the negative anode side of the battery, which are accepted via a reduction reaction on the positive cathode side of the battery. VRFBs have a specific energy of 10–35 Wh kg^{-1}, specific power of 100–166 W kg^{-1}, round-trip efficiency of 35%–85%, and a service lifetime of 15 years.[59] VRFBs provide capacity-firming and time-shift functions and not high-capacity bulk energy storage applications.

Renewable Energy Deployment System (ReEDS) Grid Mix Scenarios

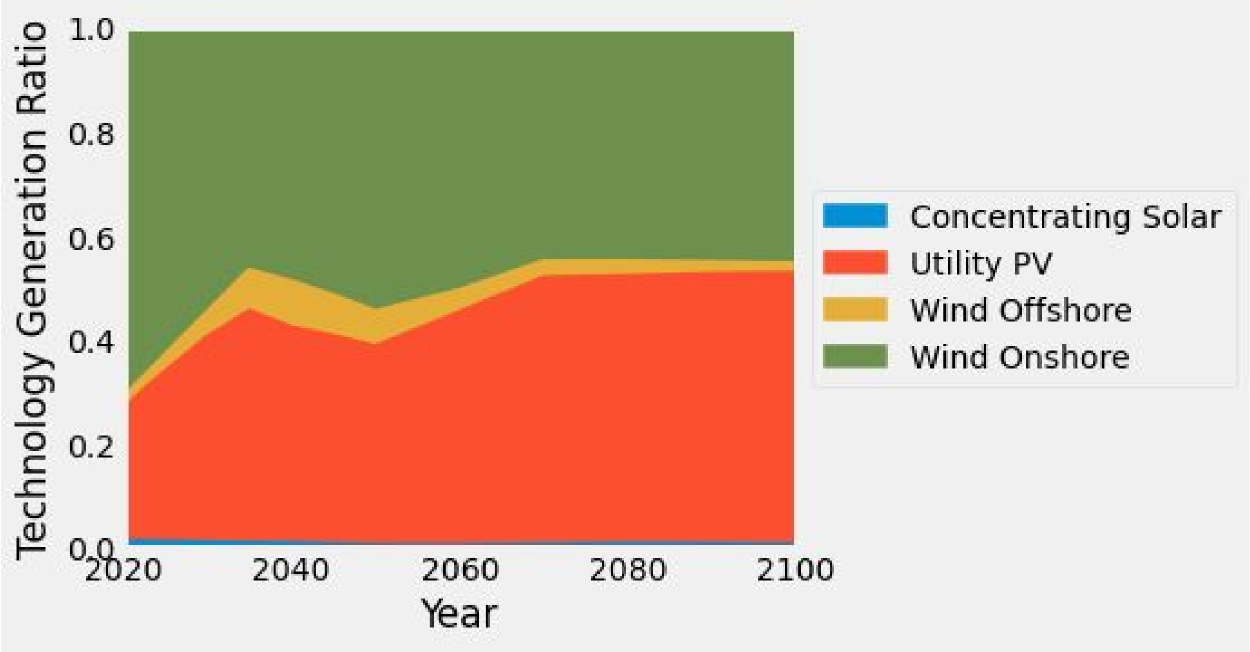

Figure S3. Projected Base Case electricity grid mix from the ReEDS model.

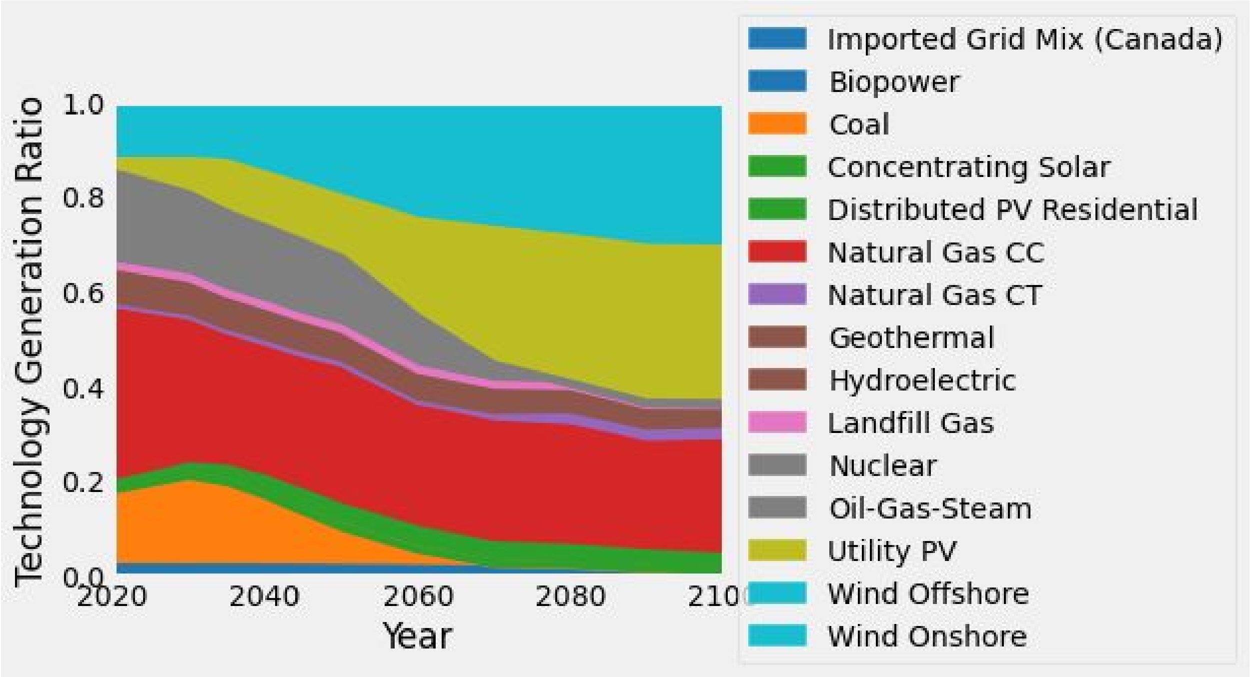

Figure S4. ReEDS results for generation technology grid mix ratios over an 80-year lifetime based on the Mid-Case 80 scenario. CC = combined cycle, CT = combustion turbine.

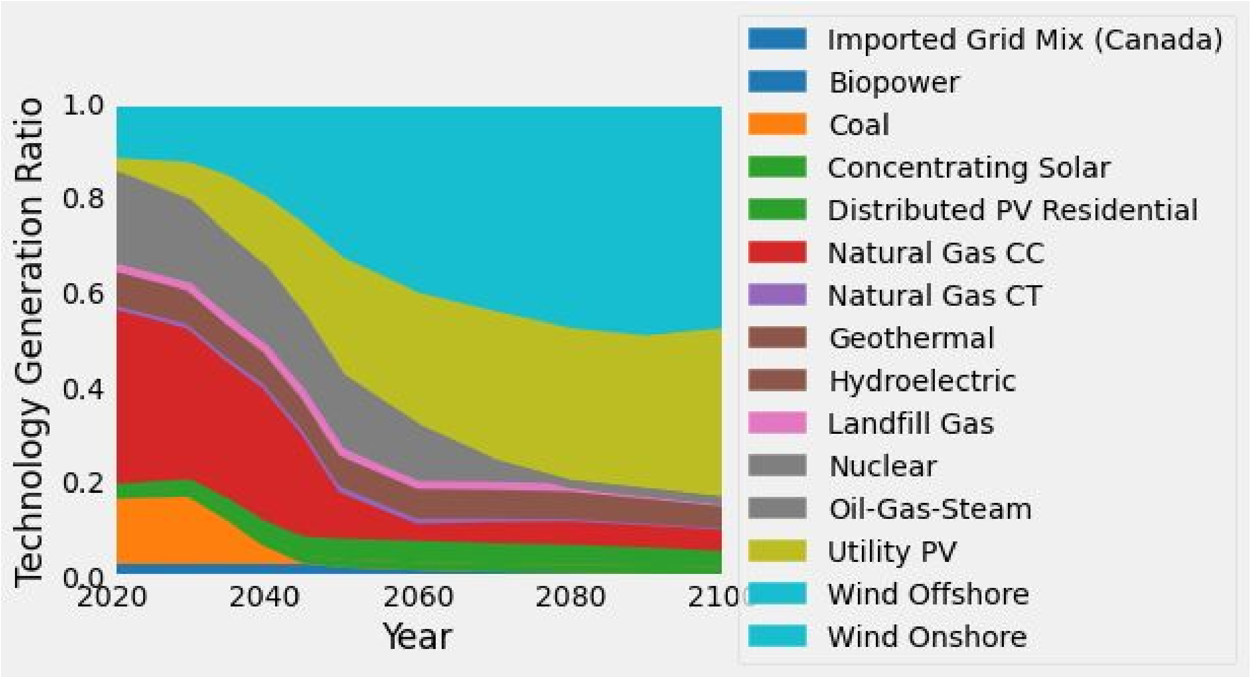

Figure S5. ReEDS results for generation technology grid mix ratios over an 80-year lifetime based on the scenario for 95% CO_2-eq reduction from 2005 levels by 2050. CC = combined cycle, CT = combustion turbine.

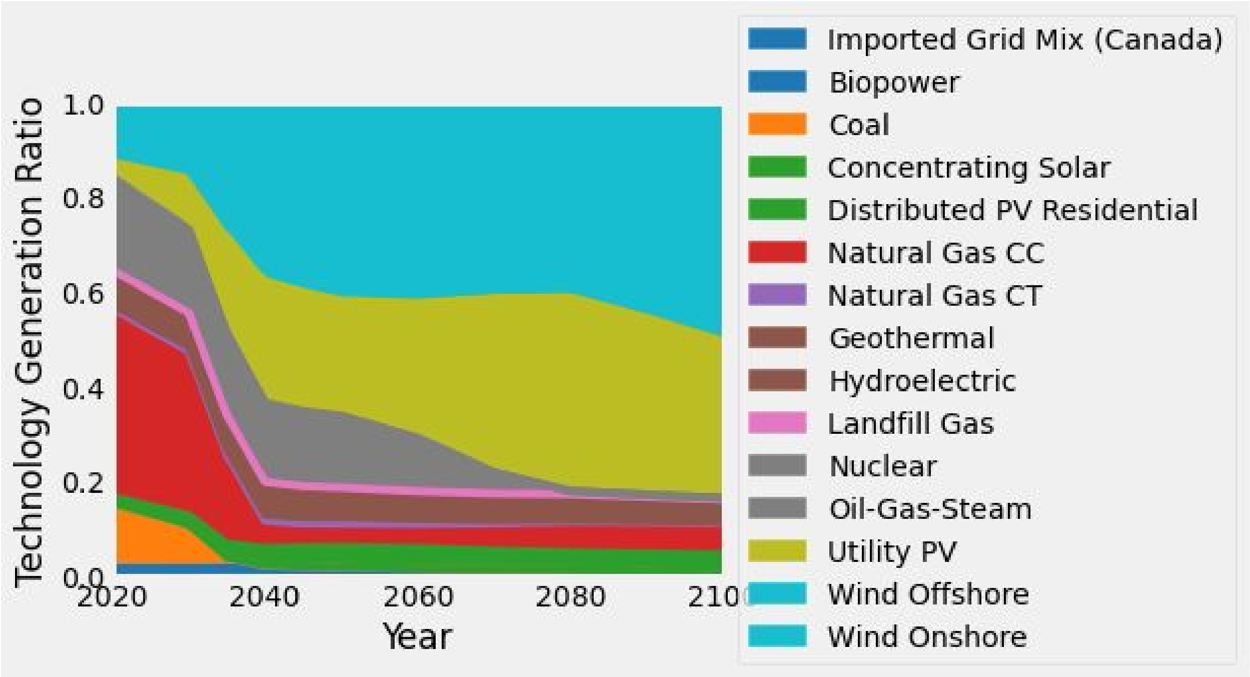

Figure S6. ReEDS results for generation technology grid mix ratios over an 80-year lifetime based on the scenario for 95% CO_2-eq reduction from 2005 levels by 2035 and 100% CO_2-eq reduction by 2050. CC = combined cycle, CT = combustion turbine.

Material Inputs

Concrete

Concrete inputs are used in several locations in a pumped hydro storage power plant. While concrete is not the primary construction material for earthfill or rockfill embankment dams, it can serve as an impermeable barrier on the upstream face of a rockfill dam, if listed as such in the project details. This concrete face is assumed to cover the entire upstream face of the dam with a thickness that varies by depth. Any dams made purely from concrete follow the listed specifications of the project and are assumed to have a 30-foot thickness (since this value is not typically listed for new PSH projects). The volume of the surge chamber is assumed to be proportional to the capacity of the PSH plant,[62] and has concrete as the main structural support.

Concrete inputs for the tunnels and powerhouse were described in a previous update and are dependent on the project specifications available as well as an estimated thickness of concrete for construction. The total volume of concrete for tunnels includes the inputs for tunnels that are not part of the main headrace or tailrace sections, including any access or vertical shafts listed in the project description. The main change made for the method of calculation for these inputs is to scale down the total concrete volume by accounting for steel reinforcement. A smaller percentage of steel reinforcement would be needed for the tunnels and a larger amount is required for the powerhouse and other internal structures, as well as the dam face and any dams made purely from concrete. There is also the addition of concrete anchors for the penstock,

which are assumed to be 40 m^3 for smaller projects and 80 m^3 for larger sites. These anchors would not require steel reinforcement. The specific flows of concrete relative to the total are shown in the figure below.

Steel

Steel is used in multiple locations within a PSH plant, primarily as reinforcement for the concrete that serves as structural support for the main sections of the plant. All concrete is assumed to be reinforced other than the penstock anchors, with steel providing 5% of the mass input for dams constructed primarily from concrete and 1% of the mass input for all other structural support concrete used. The penstock is assumed to be constructed from steel, with the total volume required determined by the specifications listed for each project. The thickness of the steel used in the penstock is based on values from literature, since there are few recent examples of PSH construction to draw from. Steel is also used as an inner layer of support in the surge chamber, on the inner wall of the main concrete structure.

Stainless steel is also the primary component of the pump/turbine that serves as one of the main pieces of electrical equipment in the powerhouse and that makes the entire PSH project possible. Each project lists the number of total pumps and turbines that will be required for construction, as well as the average (or in some cases individual) sizes of each unit. We are also assuming that each unit is a combination reversible pump and turbine, which can operate in pumping mode for moving water from the lower to the upper reservoir, or generating mode where water flows down from the upper reservoir through a turbine. This method is used from previous PSH life cycle assessments (LCAs), and the units are available in several sizes based on the needs of the project. The size of each unit determines the overall weight of both stainless steel and copper within the pump/turbine, using data supplied by an outside vendor for the weight of several units. The weights of each material were interpolated for generator sizes that did not exactly match those provided by the vendor.

The other major electrical equipment required is a transformer. The initial size was provided through a literature estimate, and sizes for different generator sizes were scaled based on the LCA results that examined several sizes of transformer.[63] Vendor data was used to determine the proportion of steel and copper used in a typical transformer, one of which is required for each project. The size is determined by the size of generator used for each project. Stainless steel and reinforced (or low-alloyed steel) are reported separately due to their distinction within Ecoinvent.

Embankment Earthfill and Rockfill

The primary dam type used in new closed-loop PSH projects in the United States is an embankment dam. These dams can be earthfill or rockfill and can vary in terms of the layers of impervious materials within the embankment and the slope required on the upstream and downstream faces of the dam. Based on the frequency of project specifications, embankment dams that do not list a material type are assumed to be rockfill dams. While in reality these dams would differ in terms of the shape and size, depending on the geology of the area and the changing needs of the project, none of these construction plans are finalized so several uniform

assumptions are made regarding the dam specifications. This includes the width of the central dam section where the embankment slopes meet, the slope of the upstream and downstream faces for earthfill and rockfill dam types, and the type of slope protection used.

Calculations for embankment inputs begins with the required volume, split up into the three main sections of the upstream and downstream faces as well as the central arc. Dam height and length are provided in each project specifications, so an assumed central arc width of 30 feet is used for all sites. Upstream and downstream slope volumes can be determined once the slope ratio is known: earthfill 1:2.5 for upstream and 1:3 for downstream, rockfill 1:1.3 for both upstream and downstream.[64] Rockfill dams have a concrete face on the upstream slope if listed in the project description, otherwise the main slope protection comes in the form of riprap added near the water's surface on the upstream slope. It is assumed that the riprap will be an average of two feet tall and cover the entire dam length in the area where the water surface meets the embankment.

Water

Water is required both as an initial construction material for the initial fill of the reservoirs as well as annual replenishment to account for evaporation and operational losses. The initial fill is determined through the reported volume of new reservoirs, as existing reservoirs, lakes, or ponds are assumed to not require additional water for operation to begin. The annual water replenishment is estimated by using the annual evaporation rate for the county where each site is located.[65] The average county evaporation rate is used as a proxy for more specific values that may be more site specific depending on the typical weather and elevation of that site. Combined with the reported surface area of each reservoir, an estimate for the water that would need to be annually replenished can be found.

Electricity

Electricity is required both for initial construction and as a major input for regular operation. Electricity for operation is used to operate the pumps and move water to the upper reservoir, which is how the energy storage takes place. The amount of electricity required depends on the overall efficiency and losses of the system starting from the point the pump begins operating and ending with the electricity produced by the generator. An overall average efficiency of 78% is used for the system, based on a literature review of existing PSH sources. The efficiency of the system depends on the specific components used and the design of the tunnels and water flow, so once construction begins on the projects used one could obtain a more specific idea of the efficiency at a given site. This value also accounts for the losses from pump to new generation; an additional 5% transmission loss is assumed for the transfer of this electricity to the grid where it will be used.

Electricity used for initial construction is a projected value in this report, since previous inputs of this metric in LCAs rely either on reported values or environmentally extended input-output (EEIO) tables. Using these methods would require waiting until construction is complete and documentation is available regarding the final inputs for the site. The estimates for sites in this report uses a metric based on the totals reported for previous PSH LCAs, as reported in megawatt-hours of electricity per megawatt capacity.

Diesel Fuel

Similar to the electricity requirements for initial construction, the diesel used to operate on-site machinery is primarily determined after construction is complete for a given site. Since none of the projects examined have completed (or begun) construction, this report uses a metric for diesel consumption based on reported values from previous PSH LCAs. The diesel used for construction, as reported in L/MW capacity, is separate from the fuel requirements for transportation of key materials to the site and is described in a separate section.

Transportation

Rail, freight, and lorry (truck) are required to transport materials to construction sites. In considering each mode of transport, assumptions are made based on various site locations to come up with average transportation distance of necessary commodities to manufacturing plants and the construction sites. Embankment dams typically make use of nearby material aggregates for construction to reduce transportation costs. After approximating each site location via GPS coordinates, most sites were found to be within 20 miles of several nearby quarries that can serve as a source for dam construction material. It is assumed that all earthfill and rockfill material is transported via lorry due to the close proximity of identified aggregate quarries.

Concrete plant locations in the United States were identified for all projects to determine the closest plant to each site location. Concrete is transported from the plant by rail to the nearest freight station for each site; afterwards the concrete is transported via lorry from freight stations to be mixed on site. The same methods for determining total transport distances of each method were also performed for reinforced steel. Stainless steel and copper are used mostly in pump/turbines, generators, and transformers. Transport of these material inputs from an international to domestic location is performed by cargo ships or freighters to a U.S. docking site, then via rail to the nearest freight station, and is finally brought to each PSH site by lorry. Manufacturing of pump/turbines, generators, and transformers is done in Tianjin, China and Taubate, Brazil. Shipping distances are estimated using *Searates* by DP World, which offers estimated distances between freight shipping ports based on common routes that would be used. Port distances were mapped from California, Texas, and New York based on locations of PSH U.S. sites considered.

Lubricating Oil and Sulfur Hexafluoride (SF_6)

Lubricating oil and SF_6 emissions are expected to occur during regular operation due to leaks, accidents, and regular use of machinery.[66] Lubricating oil is applied to moving mechanical parts in the power station regularly and is emitted to the ground and water, while SF_6 is used in electrical insulation and switches and is released during irregular operation events.

Reservoir Greenhouse Gas Emissions

Reservoirs will emit greenhouse gases (GHGs) annually due to the trapped water on a previously dry settlement. The emissions from vegetation are not expected to be large since the largest site in the west do not have large amounts of vegetation that would be removed, and larger flora does not decompose readily even after years of being submerged. The emissions can then come from

surface soil carbon and the water impediment, although these may not be as large as previous estimates imply. This annual emission should only account for the GHG emissions resulting from creation of the PSH site, excluding those that would be naturally released from the ground or nearby sources. Many previous estimates also look at reservoir emissions from dams of a natural river, as opposed to closed-loop sources where the only water flow is between the two reservoirs and have no connection to nearby rivers.

Reservoir emissions[67] are calculated per area per day, then expanded to cover the surface area of the reservoirs for each PSH site. 25% of the total emissions are attributed to the reservoir, based on observations by Prairie 2017,[68] which assumes that 75% of GHG emissions are just temporarily displaced and would be released regardless of the reservoir being constructed.

Explosives

New reservoirs are assumed to be created with explosives, as was the case for other PSH LCAs that provided an inventory as part of their reporting. The factor used for explosives is kilogram explosive per kilowatt of site capacity and is relatively consistent for each of the five reports listing this input. The average value for this input coupled with the megawatt capacity for each site is used to estimate the total amount of explosives that would be required.

Reservoir Lining

It is assumed that any new reservoirs will use some form of a geotechnical lining to reduce ground seepage from the reservoir and lessen the annual water makeup for that site. This lining is assumed to cover the entire lower surface area for any reservoir where it is deployed. While the final shape of the reservoirs has not been finalized, approximating the lower 3D surface as an ellipsoid with dimensions that match the listed volume and surface area of the reservoir provides an initial value to work from. It was found that varying the length and width of the ellipsoid did not affect the surface area substantially. The geotechnical lining would be formed from layered plastic film, typically LDPE or VLDPE, as well as a geo-woven lining. The thickness of the reservoir lining is estimated through input from an outside vendor and applying a safety margin since the reservoirs in these projects are larger than those provided by the vendor. The surface area of each reservoir is calculated individually for each newly constructed reservoir—it is assumed that existing bodies of water would not require a lining.

References

1. Federal Energy Regulatory Commission. Big Chino Valley Pumped Storage LLC; Notice of Preliminary Permit Application Accepted for Filing and Soliciting Comments, Motions To Intervene, and Competing Applications. *Fed Regist.* **2017**, *82*, 48716.

2. Federal Energy Regulatory Commission. Pumped Hydro Storage LLC; Notice of Preliminary Permit Application Accepted for Filing and Soliciting Comments, Motions To Intervene, and Competing Applications. *Fed Regist.* **2020**, *85*, 35299–35300.

3. Preliminary Permit Issued for 1,500-MW Salt River Project Indian Spring Pumped Storage Project, 2020. Hydro Review Website. https://www.hydroreview.com/regulation-and-policy/preliminary-permit-issued-for-1500-mw-salt-river-project-indian-spring-pumped-storage-project/#gref.

4. Federal Energy Regulatory Commission. Pumped Hydro Storage LLC; Notice of Preliminary Permit Application Accepted for Filing and Soliciting Comments, Motions To Intervene, and Competing Applications. *Fed Regist.* **2019**, *84*, 49722–49723.

5. Pumped Storage Projects, 2022. Federal Energy Regulatory Commission Website. https://www.ferc.gov/licensing/pumped-storage-projects.

6. Federal Energy Regulatory Commission. Notice of Preliminary Permit Application Accepted for Filing and Soliciting Comments, Motions To Intervene, and Competing Applications; Renewable Energy Aggregators. *Fed Regist.* **2019**, *84*, 72351–72352.

7. Federal Energy Regulatory Commission. Renewable Energy Aggregators, Inc.; Notice of Preliminary Permit Application Accepted for Filing and Soliciting Comments, Motions To Intervene, and Competing Applications. *Fed Regist.* **2020**, *85*, 34430.

8. Federal Energy Regulatory Commission. Pumped Hydro Storage LLC; Notice of Preliminary Permit Application Accepted for Filing and Soliciting Comments, Motions To Intervene, and Competing Applications. *Fed Regist.* **2019**, *84*, 66895.

9. Federal Energy Regulatory Commission. Renewable Energy Aggregators; Notice of Preliminary Permit Application Accepted for Filing and Soliciting Comments, Motions To Intervene, and Competing Applications. *Fed Regist.* **2020**, *85*, 36395–36396.

10. Federal Energy Regulatory Commission. Notice of Preliminary Permit Application Accepted for Filing and Soliciting Comments, Motions To Intervene, and Competing Applications; RAMM Power Group, LLC. *Fed Regist.* **2018**, *83*, 13979.

11. Application for Preliminary Permit San Vicente Pumped Storage Project, 2014. San Diego County Water Authority.

12. Federal Energy Regulatory Commission. Order Issuing Preliminary Permit and Granting Priority to File License Application, San Diego County Water Authority Project No. 14642-002. 2018. 165 FERC 62,058.

13. Federal Energy Regulatory Commission (FERC) Project No. #13123 Project Name: Eagle Mountain Pumped Storage Project (Eagle Mountain Project); FERC #13123, 2013. California Water Boards Website. https://www.waterboards.ca.gov/waterrights/water_issues/programs/water_quality_cert/eaglemtn_ferc13123.html.

14. Federal Energy Regulatory Commission. Covington Mountain Hydro, LLC; Notice of Preliminary Permit Application Accepted for Filing and Soliciting Comments, Motions To Intervene, and Competing Applications. *Fed Regist.* **2018**, *83*, 1258.

15. Federal Energy Regulatory Commission. Craig-Hayden PS, LLC; Notice of Preliminary Permit Application Accepted for Filing and Soliciting Comments, Motions To Intervene, and Competing Applications. *Fed Regist.* **2020**, *85*, 75005.

16. Cassell, Barry. FERC Issues Permit for 400-MW Pumped Storage Hydro Project in Idaho, 2015. TransmissionHub Website. https://www.transmissionhub.com/articles/2015/11/ferc-issues-permit-for-400-mw-pumped-storage-hydro-project-in-idaho.html.

17. Federal Energy Regulatory Commission. Cat Creek Energy, LLC; Notice of Successive Preliminary Permit Application Accepted for Filing and Soliciting Comments, Motions To Intervene, and Competing Applications. *Fed Regist.* **2018**, *83*, 60835–60836.

18. Federal Energy Regulatory Commission. Order Issuing Original License re GB Energy Park, LLC under P-13642. 2016. FERC Cite Number 157 FERC 62,196.

19. Gordon Butte Pumped Storage Hydro Project, 2022. GB Energy Park Website. https://www.gordonbuttepumpedstorage.com/.

20. Cassell, B. FERC Takes Comment on 250-MW Nevada Pumped Storage Project, 2014. TransmissionHub Website. https://www.transmissionhub.com/articles/2014/01/ferc-takes-comment-on-250-mw-nevada-pumped-storage-project.html.

21. Cassell, B. Gridflex Looks at 250-MW Pumped Storage Hydro Project in Nevada, 2013. TransmissionHub Website. https://www.transmissionhub.com/articles/2013/09/gridflex-looks-at-250-mw-pumped-storage-hydro-project-in-nevada.html.

22. Federal Energy Regulatory Commission. Gridflex Energy, LLC; Notice of Preliminary Permit Application Accepted for Filing and Soliciting Comments, Motions To Intervene, and Competing Applications. *Fed Regist.* **2020**, *85*, 5425–5426.

23. Federal Energy Regulatory Commission. Control Technology, Inc.; Notice of Preliminary Permit Application Accepted for Filing and Soliciting Comments, Motions To Intervene, and Competing Applications. *Fed Regist.* **2017**, *82*, 18141–18142.

24. Cassell, B. Company Updates FERC on 450-MW Pumped Storage Project in Nevada, 2014. TransmissionHub Website. https://www.transmissionhub.com/articles/2014/02/company-updates-ferc-on-450-mw-pumped-storage-project-in-nevada.html.

25. Federal Energy Regulatory Commission. Nevada PSH Energy Storage LLC; Notice of Preliminary Permit Application Accepted for Filing and Soliciting Comments, Motions To Intervene, and Competing Applications. *Fed Regist.* **2020**, *85*, 59780–59781.

26. Federal Energy Regulatory Commission. Kinetic Power, LLC; Notice of Preliminary Permit Application Accepted for Filing and Soliciting Comments, Motions To Intervene, and Competing Applications. *Fed Regist.* **2020**, *85*, 59782–59783.

27. Federal Energy Regulatory Commission. Notice of Preliminary Permit Application Accepted for Filing and Soliciting Comments, Motions To Intervene, and Competing Applications; Gridflex Energy, LLC. *Fed Regist.* **2020**, *85*, 3677.

28. Projects, Lyon Mountain Energy Storage Project FERC Project No. P-14692, no date. Serium Energy Storage Website. https://www.seriumenergystorage.com/projects.

29. Federal Energy Regulatory Commission. Albany Engineering Corporation; Notice of Preliminary Permit Application Accepted for Filing and Soliciting Comments, Motions To Intervene, and Competing Applications. *Fed Regist.* **2015**, *80*, 52750–52751.

30. Federal Energy Regulatory Commission. Merchant Hydro Developers, LLC; Notice of Preliminary Permit Application Accepted for Filing and Soliciting Comments, Motions To Intervene, and Competing Applications. *Fed Regist.* **2017**, *82*, 42680–42681.

31. Federal Energy Regulatory Commission. Notice of Preliminary Permit Application Accepted for Filing and Soliciting Comments, Motions To Intervene, and Competing Applications: New Summit Hydro, LLC. *Fed Regist.* **2014**, *79*, 24416–24417.

32. Federal Energy Regulatory Commission. Maysville PSH, LLC; Notice of Preliminary Permit Application Accepted for Filing and Soliciting Comments, Motions To Intervene, and Competing Applications. *Fed Regist.* **2020**, *85*, 80777.

33. FERC Issues Preliminary Permit to Study 1,200-MW Southeast Oklahoma Pumped Storage Project, 2019. Hydro Review Website. https://www.hydroreview.com/world-regions/ferc-issues-preliminary-permit-to-study-1-200-mw-southeast-oklahoma-pumped-storage-project/#gref.

34. Federal Energy Regulatory Commission. Southeast Oklahoma Power Corporation; Notice of Preliminary Permit Application Accepted for Filing and Soliciting Comments, Motions To Intervene, and Competing Applications. *Fed Regist.* **2018**, *83*, 60421.

35. Federal Energy Regulatory Commission. Southeast Oklahoma Power Corporation; Notice of Preliminary Permit Application Accepted for Filing and Soliciting Comments, Motions To Intervene, and Competing Applications. *Fed Regist.* **2018**, *83*, 60415–60416.

36. Federal Energy Regulatory Commission. Prineville Energy Storage LLC, Ochoco Irrigation District; Notice of Preliminary Permit Application Accepted for Filing and Soliciting Comments, Motions To Intervene, and Competing Applications. *Fed Regist.* **2016**, *81*, 62496–62497.

37. Cassell, B. FERC Grants Permit on 150-MW Prineville Pumped Storage Project, 2013. TransmissionHub Website. https://www.transmissionhub.com/articles/2013/07/ferc-grants-permit-on-150-mw-prineville-pumped-storage-project.html.

38. Bowman Dam Hydro Power, no date. City of Prineville Oregon Website. https://www.cityofprineville.com/publicworks/page/bowman-dam-hydro-power.

39. Swan Lake North Pumped Storage, 2016. Permitting Dashboard Website. https://www.permits.performance.gov/permitting-projects/swan-lake-north-pumped-storage.

40. Federal Energy Regulatory Commission. Owyhee Energy Storage, LLC; Notice of Preliminary Permit Application Accepted for Filing and Soliciting Comments, Motions To Intervene, and Competing Applications. *Fed Regist.* **2019**, *84*, 56804–56805.

41. FERC Issues Preliminary Permits for Pumped-Storage Projects in West Virginia, Oregon, 2020. Hydro Review Website. https://www.hydroreview.com/regulation-and-policy/ferc-issues-preliminary-permits-for-pumped-storage-projects-in-west-virginia-oregon/#gref.

42. Federal Energy Regulatory Commission. Peak Hour Power, LLC; Notice of Preliminary Permit Application Accepted for Filing and Soliciting Comments, Motions To Intervene, and Competing Applications. *Fed Regist.* **2018**, *83*, 29781.

43. Federal Energy Regulatory Commission. Grid Balance Hydropower, LLC; Notice of Preliminary Permit Application Accepted for Filing and Soliciting Comments, Motions To Intervene, and Competing Applications. *Fed Regist.* **2019**, *84*, 45139–45140.

44. Federal Energy Regulatory Commission. Notice of Preliminary Permit Application Accepted for Filing and Soliciting Comments, Motions To Intervene, and Competing Applications; Flat Canyon Hydro, LLC. *Fed Regist.* **2018**, *83*, 17174.

45. Goldendale Energy Storage Project – A Community Driven Approach to 100% Renewable Energy, no date. Goldendale Energy Storage Website. https://goldendaleenergystorage.com/index.html.

46. Federal Energy Regulatory Commission. Filing Description for Accession Number 20191216-5010, Draft License Application for the Goldendale Energy Storage Project, FERC No. 14861. FERC eLibrary Website. https://elibrary.ferc.gov/eLibrary/filelist?accession_number=20191216-5010.

47. Federal Energy Regulatory Commission. Badger Mountain Hydro, LLC; Notice of Declaration of Intention and Soliciting Comments, Protest, and Motions To Intervene. *Fed Regist.* **2020**, *85*, 23514.

48. Federal Energy Regulatory Commission. Black Canyon Hydro, LLC; Notice of Preliminary Permit Application Accepted for Filing and Soliciting Comments, Motions To Intervene, and Competing Applications. *Fed Regist.* **2016**, *81*, 44606.

49. Pohl, M. M. Bringing down our dams: Trends in American dam removal rationales. *J. Am. Water Resour. Assoc.* **2007**, *38* (6), 1511–1519.

50. American Rivers Dam Removal Database, 2017. Figshare Website. https://figshare.com/articles/dataset/American_Rivers_Dam_Removal_Database/5234068/2 (accessed June 9, 2022).

51. Oliveira, L.; Messagie, M.; Mertens, J.; Laget, H.; Coosemans, T.; Van Mierlo, J. Environmental performance of electricity storage systems for grid applications, a life cycle approach. *Energy Convers. Manage.* **2015**, *101*, 326–335.

52. Schmidt, T. S.; Beuse, M.; Zhang, X.; Steffen, B.; Schneider, S. F.; Pena-Bello, A.; Bauer, C.; Parra, D. Additional emissions and cost from storing electricity in stationary battery systems. *Environ. Sci. Technol.* **2019**, *53* (7), 3379–3390.

53. Baumann, M.; Peters, J. F.; Weil, I. M.; Grunwald, A. CO2 footprint and life-cycle costs of electrochemical energy storage for stationary grid applications. *Energy Technology* **2016**, *5* (7), 1071–1083.

54. Abdon, A.; Zhang, X.; Parra, D.; Patel, M. K.; Bauer, C.; Worlitschek, J. Techno-economic and environmental assessment of stationary electricity storage technologies for different time scales. *Energy* **2017**, *139*, 1173–1187.

55. Hiremath, M.; Derendorf, K.; Vogt T. Comparative life cycle assessment of battery storage systems for stationary applications. *Environ. Sci. Technol.* **2015**, *49* (8), 4825–4833.

56. Bouman, E. A.; Oberg, M. M.; Hertwich, E. G. Environmental impacts of balancing offshore wind power with compressed air energy storage (CAES). *Energy* **2016**, *95*, 91–98.

57. *EPRI-DOE Handbook of Energy Storage for Transmission and Distribution Applications*; Final Report 1001834. EPRI: Palo Alto, and U.S. Department of Energy: Washington, D.C., 2003.

58. Chen, T.; Jin, Y.; Lv, H.; Yang, A.; Liu, M.; Chen, B.; Xie, Y.; Chen, Q. Applications of lithium-ion batteries in grid-scale energy storage systems. *Trans. Tianjin Univ.* **2020**, *26*, 208–217.

59. Kebede, A. A.; Kalogiannis, T.; Van Mierlo, J.; Berecibar, M. A comprehensive review of stationary energy storage devices for large scale renewable energy sources grid integration. *Renewable Sustainable Energy Rev.* **2022**, *159*, 112213.

60. Enos, D. G. Chapter 3 – Lead-Acid Batteries for Medium- and Large-Scale Energy Storage. In. *Woodhead Publishing Series in Energy, Advances in Batteries for Medium and Large-Scale Energy Storage*; Menictas, C.; Skyllas-Kazacos, M.; Lim, T. M., Eds.; Woodhead Publishing, 2015; pp 57–71.

61. Dufo-Lopez, R.; Cortes-Arcos, T.; Artal-Servil, J. S.; Bernal-Augustin, J. L. Comparison of lead-acid and Li-ion batteries lifetime prediction models in stand-alone photovoltaic systems. *Appl. Sci.* **2021**, *11* (3), 1099.

62. Sandvåg, S.U. (2016) Surge tank atlas for hydropower plants. Norwegian University of Science and Technology Department of Hydraulic and Environmental Engineering. (M.Sc. Thesis) Available at https://core.ac.uk/download/pdf/154676617.pdf

63. Santos Jorge, R.; Hawkins, T. R.; Hertwich, E. G. Life cycle assessment of electricity transmission and distribution—part 2: transformers and substation equipment. *Int. J. Life Cycle Assess.* **2012**, *17*, 184–191.

64. U.S. Army Corps of Engineers. General design and construction considerations for earth and rock-fill dams: Engineer manual. **2004**, EM 1110-2-2300.

65. Sanford, W. E.; Selnick, D. L. Estimation of evapotranspiration across the conterminous United States using a regression with climate and land-cover data. *J. Am. Water Resources Assoc.* **2012**, *49* (1), 217–230.

66. Vattenfall. Certified environmental product declaration of electricity from Vattenfalls' Nordic Hyrdropower. Retrieved from www.vattenfall.com.

67. World Bank Group. Greenhouse gases from reservoirs caused by biogeochemical processes. **2017**. Available at

https://documents1.worldbank.org/curated/en/739881515751628436/pdf/Greenhouse-gases-from-reservoirs-caused-by-biogeochemical-processes.pdf.

68. Prairie, Y. T.; Alm, J.; Beaulieu, J.; Barros, N.; Battin, T.; Cole, J.; del Giorgio, P.; DelSontro, T.; Guerin, F.; Harby, A.; Harrison, J.; Mercier-Blais, S.; Serca, D.; Sobek, S.; Vachon, D. Greenouse gas emissions from freshwater reservoirs: what does the atmosphere see? *Ecosystems* **2018**, *21* 1058–1071.
